# Supplementary material for: Conditional chemoconnectomics (cCCTomics) as a strategy for efficient and conditional targeting of chemical transmission
Source: eLife. 2024 Apr 30;12:RP91927. doi: 10.7554/eLife.91927 (PMC11060718; doi:10.7554/eLife.91927)
Supplement: Figure 3—source data 3. [file elife-91927-fig3-data3.zip › Figure 3 source data3 part 1.zip!/Figure 3-Source data 3-part1/1063f3-1-CRISPResso_Report_kboyr13q.zip!/CRISPResso_on_1063f3_3/1a.Read_barplot.pdf]

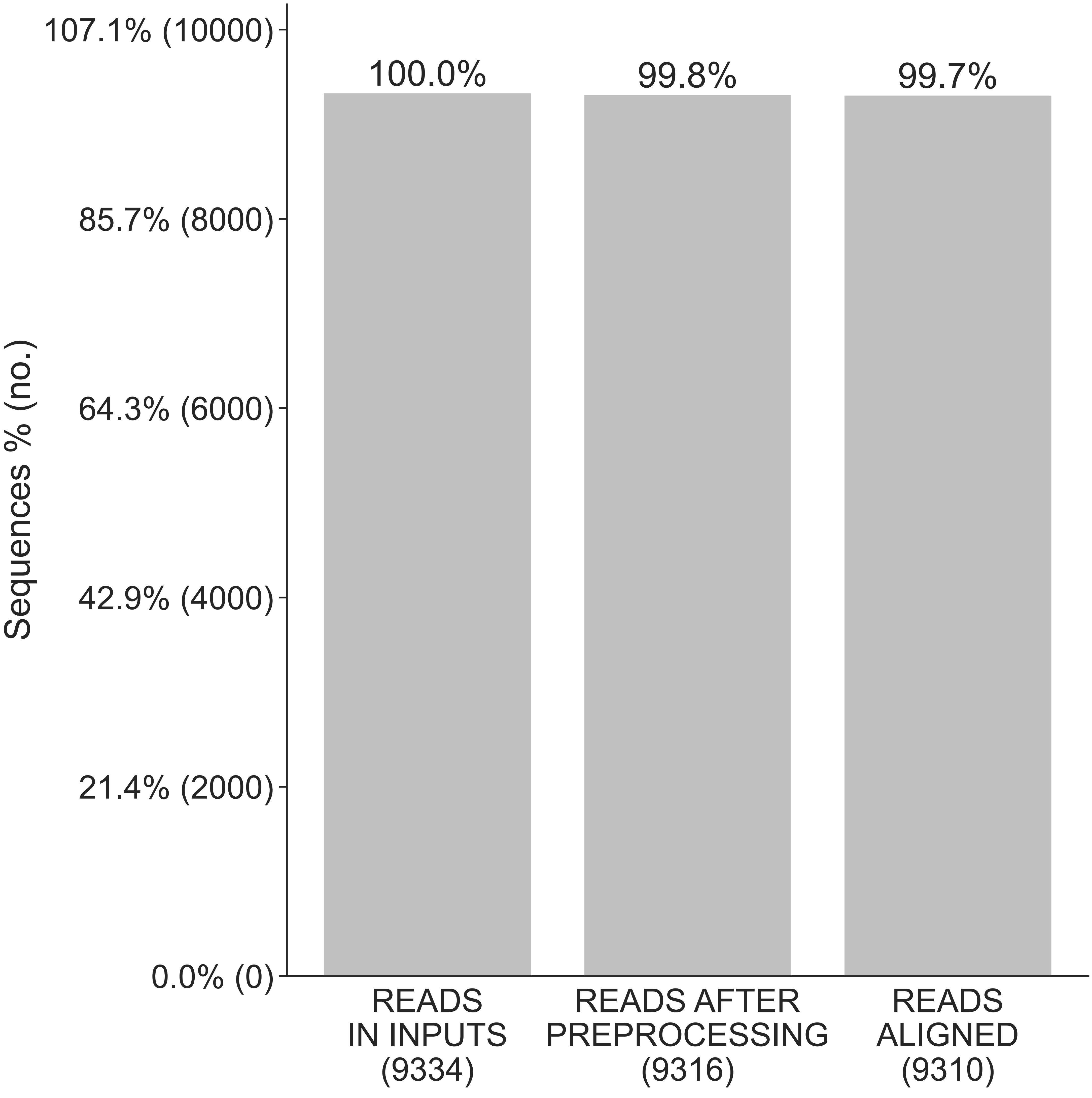
Sequences % (no.)
107.1% (10000)
85.7% (8000)
64.3% (6000)
42.9% (4000)
21.4% (2000)
0.0% (0)
100.0%
99.8%
99.7%
READS
IN INPUTS
(9334)
READS AFTER
PREPROCESSING
(9316)
READS
ALIGNED
(9310)
